# LncRNA ST7-AS1, by regulating miR-181b-5p/KPNA4 axis, promotes the malignancy of lung adenocarcinoma

**DOI:** 10.1186/s12935-020-01652-7

**Published:** 2020-12-17

**Authors:** Rong-Hang Hu, Zi-Teng Zhang, Hai-Xiang Wei, Lu Ning, Jiang-Shan Ai, Wen-Hui Li, Heng Zhang, Shao-Qiang Wang

**Affiliations:** 1 Department of Thoracic Surgery, Affiliated Hospital of Jining Medical University, Jining Medical University, No. 89, GuHuai Road, Jining, 272029 Shandong People’s Republic of China; 2 Medical College of Qingdao University, Qingdao, 266071 People’s Republic of China; 3 Department of Thoracic Surgery, Xiangya Hospital, Central South University, No. 87, Xiangya Road, Changsha, 410008 People’s Republic of China

**Keywords:** ST7-AS1, Lung adenocarcinoma, Epithelial-mesenchymal transition, miR-181b-5p, KPNA4

## Abstract

**Background:**

Growing evidence suggests that suppressor of tumorigenicity 7 antisense RNA 1 (ST7-AS1) is an oncogenic long noncoding RNA (lncRNA). However, little is known on its clinical significance, biological functions, or molecular mechanisms in lung adenocarcinoma (LUAD).

**Methods:**

The expression of ST7-AS1 and miR-181b-5p were examined by qRT-PCR. The correlations between ST7-AS1 level and different clinicopathological features were analysed. In vitro, LUAD cells were examined for cell viability, migration and invasion by MTT, wound healing and Transwell assay, respectively. Epithelial-mesenchymal transition (EMT) biomarkers were detected by Western blot. The regulations between ST7-AS1, miR-181b-5p, and KPNA4 were examined by luciferase assay, RNA immunoprecipitation, RNA pulldown. Both gain- and loss-of-function strategies were used to assess the importance of different signalling molecules in malignant phenotypes of LUAD cells. The in vivo effect was analysed using the xenograft and the experimental metastasis mouse models.

**Results:**

ST7-AS1 was upregulated in LUAD tissues or cell lines, correlated with tumours of positive lymph node metastasis or higher TNM stages, and associated with shorter overall survival of LUAD patients. ST7-AS1 essentially maintained the viability, migration, invasion, and EMT of LUAD cells. The oncogenic activities of ST7-AS1 were accomplished by sponging miR-181b-5p and releasing the suppression of the latter on KPNA4. In LUAD tissues, ST7-AS1 level positively correlated with that of KPNA4 and negatively with miR-181b-5p level. In vivo, targeting ST7-AS1 significantly inhibited xenograft growth and metastasis.

**Conclusions:**

ST7-AS1, by regulating miR-181b-5p/KPNA4 axis, promotes the malignancy of LUAD cells. Targeting ST7-AS1 and KPNA4 or up-regulating miR-181b-5p, therefore, may benefit the treatment of LUAD.

## Background

Lung cancer remains the cancer with the highest mortality, non-small cell lung cancer (NSCLC) still accounts for approximately 85% of all new lung cancer cases [1, 2]. Lung adenocarcinoma (LUAD), a subtype of NSCLC, accounts for almost 50% of NSCLC, and the prognosis for patients with LUAD is generally poor [3]. Due to tumour metastasis and recurrence, the average 5-year survival rate of LUAD patients is very low [4]. Epithelial-mesenchymal transition (EMT), a biological process whereby tumour cells transform from epithelial to mesenchymal phenotypes, is controlled by several transcription factor families and a driving force for metastatic dissemination of tumour cells, the primary cause of cancer-related deaths [5–7]. Therefore, understanding mechanisms underlying LUAD carcinogenesis and metastasis including EMT will aid the development of novel, effective therapies for LUAD patients.

Long non-coding RNAs (lncRNAs) are RNA molecules with transcripts longer than 200 nucleotides and limited coding potential. LncRNAs present diverse biological functions and work within the cells to regulate different processes through myriad molecular mechanisms [8]. Emerging evidence suggests that aberrant lncRNAs participate in LUAD development and progression [9]. For example, lncRNA CASC2 inhibits cell proliferation and migration of LUAD cells via miR-4735‐3p/mTOR axis [10]. lncRNA DGCR5 promotes LUAD progression by inhibiting miR-22-3p [4]. Therefore, identifying molecular mechanisms of LUAD-associated lncRNAs is of great significance to better understand cancer progression and establish effective treatment of LUAD. The lncRNA suppressor of tumorigenicity 7 antisense RNA 1 (ST7-AS1) is the antisense transcript for ST7. Recent studies suggest that ST7-AS1 is an oncogenic lncRNA in both gastric cancer and laryngeal squamous cell carcinoma (LSCC) [11, 12]. However, little is known on the clinical status, biological activities, or molecular mechanisms of ST7-AS1 in LUAD.

MicroRNAs (miRNAs) are another class of non-coding RNA molecules that are 22 nucleotides in length. By binding to the 3′ untranslated region (3′UTR) of target genes, miRNAs negatively regulate gene expression post-transcriptionally [13, 14]. During cancer development, miRNAs regulate carcinogenesis and metastasis by altering oncogenic and/or tumour suppressor gene pathways [15–20]. The expression of miR-181b-5p (also named miR-181b) is aberrant in serum and tissue of NSCLC patients, which is a potential prognostic marker [21]. However, the molecular mechanism of miR-181b-5p in the control of LUAD tumorigenesis and EMT has not yet been fully reported.

In this study, we examined the clinical relevance and biological functions of lncRNA ST7-AS1 in LUAD. We found that ST7-AS1 was up-regulated in LUAD tissues, associated with tumours demonstrating more aggressive growth and shorter overall survival of LUAD patients. Invitro, ST7-AS1 promoted multiple malignant phenotypes of LUAD cells, which were mediated by sponging miR-181b-5p and subsequently up-regulating karyopherin subunit alpha 4 (KPNA4). In vivo, targeting ST7-AS1 inhibited xenograft growth and metastatic spread of LUAD cells. Therefore, targeting ST7-AS1/miR-181b-5p/KPNA4 axis may provide novel strategies for treating LUAD.

## Methods

### Human tissues

This study was reviewed and approved by the Ethics Committee of Jining Medical University. A cohort of 48 matching pairs of cancer tissues and adjacent tumor-free lung tissues were obtained during surgery from LUAD patients admitted into our hospital. TNM staging was established based on the Eighth Edition of TNM Classification for lung cancer [22]. All isolated human tissues were immediately frozen in liquid nitrogen for expressional analysis. All patients signed the informed consent to participate in this study.

### Cell lines and cell culture

Human normal bronchial epithelial cell lines 16HBE (PCS-300-010) and LUAD cell lines NCI-H1975 (CRL-5908), NCI-H1299 (CRL-5803), A549 (CCL-185) and CALU-3 (HTB-55) were purchased from the American Type Culture Collection (ATCC; Manassas, VA, USA) in June 2018. Another LUAD cell line PC-9 was obtained from Hunan Engineering Research Centre for Pulmonary Nodules Precise Diagnosis and Treatment at Xiangya Hospital of Central South University as a gift. Cells were cultured at 37 °C in humidified air with 5% CO_2_ in Dulbecco’s Modified Eagle’s Medium (DMEM) containing 10% foetal calf serum (FCS) and 1% penicillin/streptomycin (all from Gibco, USA).

### Plasmid construction and cell transfection

miR-181b-5p mimics or miR-181b-5p inhibitors and their respective non-targeting sequences (negative control, NC) were synthesized and purchased from GenePharma (Shanghai, China) and were transfected into target cells with Lipofectamine 2000 (Invitrogen, USA). Four short-hairpin ST7-AS1 (sh-ST7-AS1) plasmids and the corresponding control shRNA plasmid (sh-NC) were synthesized and purchased from Geenseed Biotech Co. (Guangzhou, China) and were used for ST7-AS1 knockdown. PcDNA3.1-ST7-AS1 (p-ST7-AS1), pcDNA3.1-KPNA4 (p-KPNA4) and empty pcDNA3.1 vector (p-NC) were synthesized and purchased from Geenseed Biotech Co. (Guangzhou, China) and were used for overexpression of ST7-AS1 and KPNA4. Transient transfection was achieved using Lipofectamine 2000 (Invitrogen, USA) following the manufacturer’s protocol. RNA and protein assays were performed at 48 h after the transfection.

### RNA extraction and quantitative real-time PCR (qRT-PCR)

Total RNA was extracted using TRIzol (Invitrogen, USA). Reverse transcription and qRT-PCR was performed using QuantiTect SYBR Green RT-PCR Kit (Qiagen, USA) and gene-specific primers listed as below. The relative expression of a target gene was calculated using the comparative Ct method and normalized to either GAPDH (for mRNA or lncRNA) or U6 (for miRNA).

ST7-AS1-F 5′-TGGGGTAACTCAAAAAGCCTG-3′

ST7-AS1-R 5′-GGTTCATACCAGCCCTGTCC-3′

Vimentin-F 5′-CTGCCAACCGGAACAATGAC-3′

Vimentin-R 5′-CATTTCACGCATCTGGCGTT-3′

Twist1-F 5′-TCAAGAGGTCGTGCCAATCA-3′

Twist1-R 5′-TTGCAGGCCAGTTTGATCCC-3′

E-cadherin-F 5′-TACCCTGGTGGTTCAAGCTG-3′

E-cadherin-R 5′-CAAAATCCAAGCCCGTGGTG-3′

KPNA4-F 5′-AGCTTGGCTCATGGGATCTG-3′

KPNA4-R 5′-ATTCTCACTCGCACCCACTC-3′

GAPDH-F 5′-AGGTCGGTGTGAACGGATTTG-3′

GAPDH-R 5′-GGGGTCGTTGATGGCAACA-3′

U6-F 5′-CGCTTCGGCAGCACATATACTA-3′

U6-R 5′-CGCTTCACGAATTTGCGTGTCA-3′

miR-181b-5p-F 5′-AACATTCATTGCTGTCGGTGGGT-3′

miR-181b-5p-R 5′-GCGAGCACAGAATTAATACGAC-3′.

### Cell viability assay

Cells viability was measured using Cell Proliferation Kit I (MTT; Sigma) following the manufacturer’s instructions. Briefly, cells were cultured in 96-well plates for indicated time periods. After incubating with MTT solution (5 mg/mL in PBS) at 37 ºC for a further 3 h, viable cells were detected using DMSO followed by reading OD570 of the plate with a Microplate Reader (Bio-Rad, USA).

### Wound healing assay

To assess cell migration, a scratch was made across the confluent layer of target cells using a sterile pipette tip. The wound was then imaged immediately (0 h) and after 24 h, respectively. The width (W) of the scratch was measured and cell migration was calculated as (W_0 h_ – W_24 h)_/W_0 h_ × 100%.

### Transwell invasion assay

Cell invasion was examined using Transwell invasion chambers (Corning, USA). PC-9 or A549 cells in serum-free DMEM were seeded into the top well, and serum-containing medium into the lower chamber. After 48 h, cells in the upper chamber were removed using a cotton swab. Cells attached to the bottom side of the membrane were fixed with methanol, stained with 0.1% crystal violet, and imaged under a microscope. The number of invaded cells from five random fields was counted for each sample.

### Luciferase reporter assay

The predicted miR-181-5p-binding sequence of ST7-AS1 with miR-181b-5p and that in the 3′ UTR of KPNA4 gene were either mutated (MUT) or not (WT), and cloned downstream of the firefly luciferase gene in the pmirGLO vector (Promega, USA). Then the reporter vector was co-transfected into A549 and PC-9 cells with mimics NC or miR-181b-5p mimics using Lipofectamine 3000 (Invitrogen, USA). After 48 h, firefly luciferase activity was measured and normalized to the Renilla luciferase activity (transfection control) using the dual-Luciferase Reporter Assay System (Promega, USA) according to the manufacturer’s instructions.

### RNA immunoprecipitation (RIP) assay

RIP was performed using the Magna RIP RNA-Binding Protein Immunoprecipitation Kit (Millipore, USA) according to instructions from the manufacturer. Briefly, Cell lysates were incubated with the anti‐Ago2 (#2897, Cell Signaling Technology) or normal anti-rabbit IgG (Cell Signaling Technology) followed by magnetic beads. RNA immunoprecipitated with the antibody was detected by RT-PCR. Target RNA detected from total RNA was used as the input control. The RNA binding with IgG was used as the negative control.

### RNA pull-down assay

The biotinylated RNA pull-down assay was performed as described earlier [23] with minor modifications. Briefly, ST7-AS1-binding sequence was amplified using a forward primer containing T7 RNA polymerase promoter sequence (T7) at the 5′ end. Then biotinylated RNA was prepared through in vitro transcription using MEGAshortscript™ T7 Kit (Thermo Fisher Scientific, USA). After purification, biotinylated RNA probe was incubated with cell lysates of either A549 or PC-9 cells. After treating the pull-down products with RNase-free DNase I (Roche, USA), the detection of miR-181-5p was performed using qRT-PCR.

### Western blot analysis

Whole cell lysates were prepared using IP lysis buffer (Thermo Fisher Scientific, USA) containing protease and phosphatase inhibitors (Sigma, USA). After electrophoresis through 10% SDS-PAGE, all proteins were transferred to PVDF membranes and incubated with the following primary antibodies (all from Abcam, USA) at 4 ºC overnight: anti-E-cadherin (#ab1416, 1:1000), anti-vimentin (#ab8978, 1:2000), anti-Twist1 (#ab50581, 1:2000), anti-KPNA4 (#ab6039, 1:1000) and anti-β-actin (#ab8226, 1:5000). After three washes in TBST buffer containing 0.1% Tween 20, the membranes were incubated with HRP-conjugated anti-mouse or rabbit IgG (1:5000, Sigma) for at room temperature 1 h. Target proteins were detected using the enhanced chemiluminescence substrate (ECL, Millipore, USA) and analysed with ImageJ software.

### In vivo experiments and histological analysis

Animal protocols used in this study were pre-approved by the Institutional Animal Care and Use Committee of Jining Medical University. Athymic nude mice of four to six weeks old were ordered from Shanghai Laboratory Animal Center (Shanghai, China) and housed in a specific-pathogen-free facility. To establish xenografts, sh-NC or sh-ST7-AS1 PC-9 cells were injected subcutaneously into the dorsal flank of each mouse (N = 4 mice/group) on day 0. Starting from day 5, xenograft growth was monitored by measuring the length (L) and width (W) with a caliper every five days, and the tumor volume (V) calculated as: V = 0.5 × L × W^2^. On day 30 after the inoculation of LUAD cells, all mice were euthanized, with xenograft tumors isolated and processed for further analysis.

To assess the metastatic spread of LUAD cells in vivo, sh-NC or sh-ST7-AS1 PC-9 cells were injected through the tail vein into each mouse (N = 4 mice/group) on day 0. On day 30, all mice were euthanized, with the lungs isolated, imaged, and prepared into 4-µm thick tissue sections. Hematoxylin and eosin (HE) staining was performed using HE staining kit (Vector Labs, Burlingame, CA, USA) according to the manufacturer’s instructions.

### Statistical analysis

Quantitative results were presented as the mean ± standard deviation (mean ± SD). Statistical analysis was performed using GraphPad Prism 6 software (GraphPad, USA). Statistical differences between two groups were analysed using one-way ANOVA followed by Newman-Keuls post hoc test. Two-tailed Student’s *t*-test was used for paired comparisons. Survival curve was graphed using Kaplan–Meier method. *P* < 0.05 was considered statistically significant.

## Results

### LncRNA ST7-AS1 is up-regulated in LUAD tissues and cells, and associated with worse overall survival of cancer patients

**Fig. 1:**
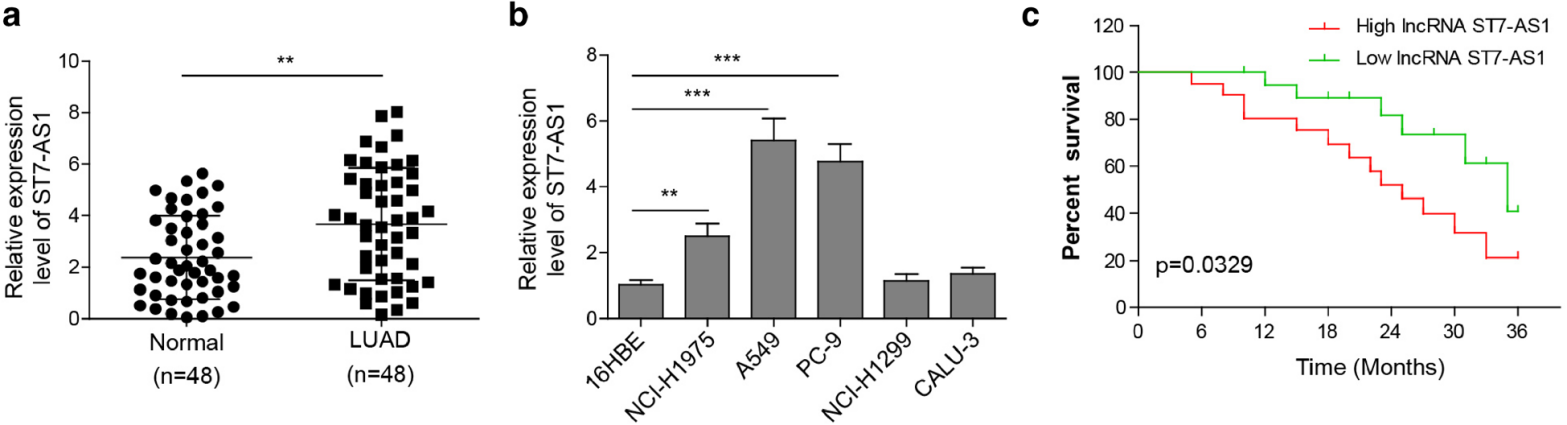
ST7-AS1 expression is elevated in LUAD tissues and cells, and associated with worse prognosis of LUAD patients. **a** ST7-AS1 expression was measured by RT-qPCR and compared between 48 pairs of LUAD tissues and normal lung tissues. **b** ST7-AS1 expression in indicated cell lines was measured by RT-qPCR. **c** The association between ST7-AS1 level and overall survival of LUAD patients was examined using Kaplan Meier analysis. **P < 0.01; ***P < 0.001

Minimal information is available for the clinical or biological importance of ST7-AS1 in LUAD. To address this question, we first compared the expression of ST7-AS1 between 48 pairs of LUAD tissues and matching tumour-free lung tissues, and found that it was markedly higher in the tumours than in normal tissues (Fig. 1a). Furthermore, we examined its expression in a panel of five different LUAD cell lines: NCI-H1975, PC-9, A549, NCI-H1299, and CALU-3. When compared to the normal bronchial epithelial cells 16HBE, the expression of ST7-AS1 was significantly upregulated in PC-9 and A549 cells compared with 16HBE cells (Fig. 1b), suggesting the heterogeneity of ST7-AS1 in LUAD cell lines. Correlation analysis between ST7-AS1 and different clinicopathological features showed that higher ST7-AS1 expression was positively correlated with tumors that metastasized to lymph nodes (*P* = 0.007), those with advanced TNM stages (stage IIB or III, IV to I or IIA; *P* = 0.019; Table 1), but reversely with the overall survival of cancer patients (Fig. 1c), suggesting that higher ST7-AS1 expression could be an indicator for LUAD of advanced malignancy and metastasis, as well as worse prognosis. The data indicated that ST7-AS1 had a potential carcinogenic effect in LUAD.

**Table 1:** Correlation between lncRNA ST7-AS1 expression and clinical pathological characteristic in LUAD patients (n = 48)

Parameters	Number	lncRNA ST7-AS1		P value
		Low (n = 24)	High (n = 24)	
Age				0.247
< 60	22	13	9	
≥ 60	26	11	15	
Gender				0.773
Male	25	12	13	
Female	23	12	11	
Tumor size (cm)				0.768
< 3	19	9	10	
≥ 3	29	15	14	
Lymph-node metastasis				0.007**
Negative	15	11	4	
Positive	33	13	20	
TNM				0.019*
I−IIA	20	14	6	
IIB−IV	28	10	18	

*P < 0.05; **P < 0.01

### LncRNA ST7-AS1 is essential for maintaining multiple malignant phenotypes of LUAD cells

**Fig. 2:**
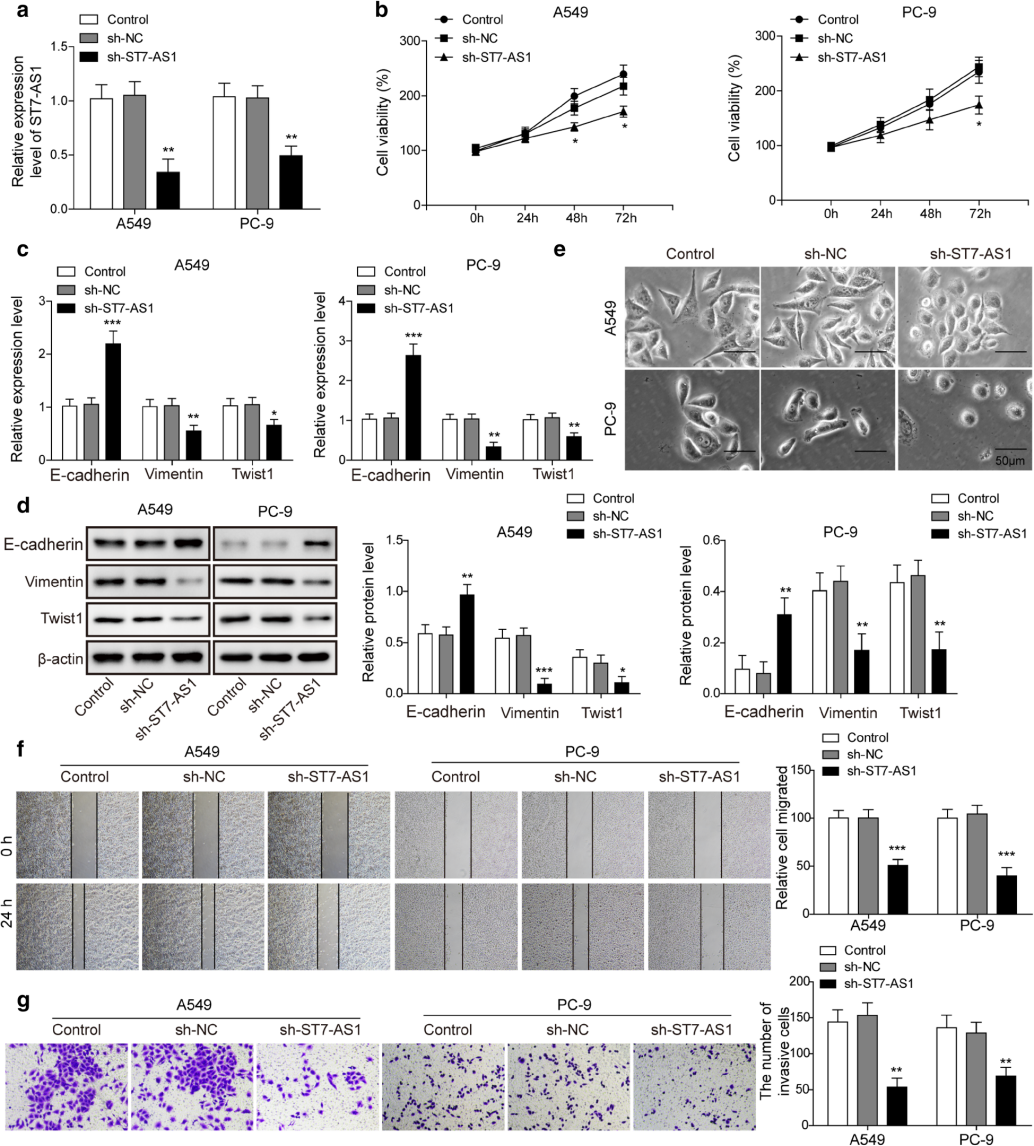
ST7-AS1 is essential for maintaining multiple malignant phenotypes of LUAD cells. **a** ST7-AS1 level in A549 (left panel) and PC-9 cells non-transfected, transfected with sh-NC or sh-ST7-AS1 was measured by RT-qPCR. **b** MTT assay was performed on indicated cells at 24 h and 48 h after transfection, respectively. **c** The expressions of EMT-related biomarkers, E-cadherin, vimentin, and Twist1 in indicated A549 and PC-9 cells were measured by RT-qPCR. **d** The protein levels of EMT-related biomarkers, E-cadherin, vimentin, and Twist1 in indicated A549 and PC-9 cells were measured by Western blot assay. **e** Indicated A549 and PC-9 cells at 72 h after transfection was imaged under a phase-contrast microscope. **f** The migration of indicated A549 and PC-9 cells was assessed by wound healing migration assay. **g** The invasion of indicated A549 and PC-9 cells was assessed by Transwell assay. Representative images of invaded cells were shown on the left and the number of invaded cells quantified on the right. *P < 0.05; **P < 0.01; ***P < 0.001

To understand the biological functions of ST7-AS1 in LUAD, we chose PC-9 and A549 cells, where ST7-AS1 was significantly up-regulated (Fig. 1c), as our model system and applied loss-of-function strategy, where both cells were transfected with sh-ST7-AS1 to reduce the endogenous ST7-AS1 level. When compared to non-transfected or sh-NC-transfected cells, ST7-AS1 was successfully knocked down in both cell lines (Fig. 2a). Viability assay revealed that sh-ST7-AS1 potently reduced the viability of both PC-9 and A549 cells (Fig. 2b). When focusing on three well-demonstrated EMT-related biomarkers, E-cadherin, vimentin and Twist1 [24], we found that sh-ST7-AS1 significantly boosted the expression of E-cadherin, but reduce that of Vimentin and Twist 1, on both the mRNA (Fig. 2c) and the protein (Fig. 2d) levels. Correspondingly, we observed that non-transfected and sh-NC-transfected (both PC-9 and A549) cells were dispersed in culture and assumed elongated, spindle-like mesenchymal morphologies, while sh-ST7-AS1 cells were cuboid cells that clustered together (Fig. 2e), indicating that knockdown of ST7-AS1 inhibited EMT progression in LUAD cells. Furthermore, we evaluated the effects of knocking down ST7-AS1 on cell migration and invasion. Results from wound healing and Transwell assays demonstrated that ST7-AS1 knockdown significantly reduced the migratory and invasive capabilities of both PC-9 and A549 cells (Fig. 2f and g). Collectively, these data reveal the essential roles of ST7-AS1 in maintaining multiple malignant phenotypes, including viability, EMT, migration and invasion of LUAD cells.

### LncRNA ST7-AS1 directly targets the expression of miR-181b-5p

**Fig. 3:**
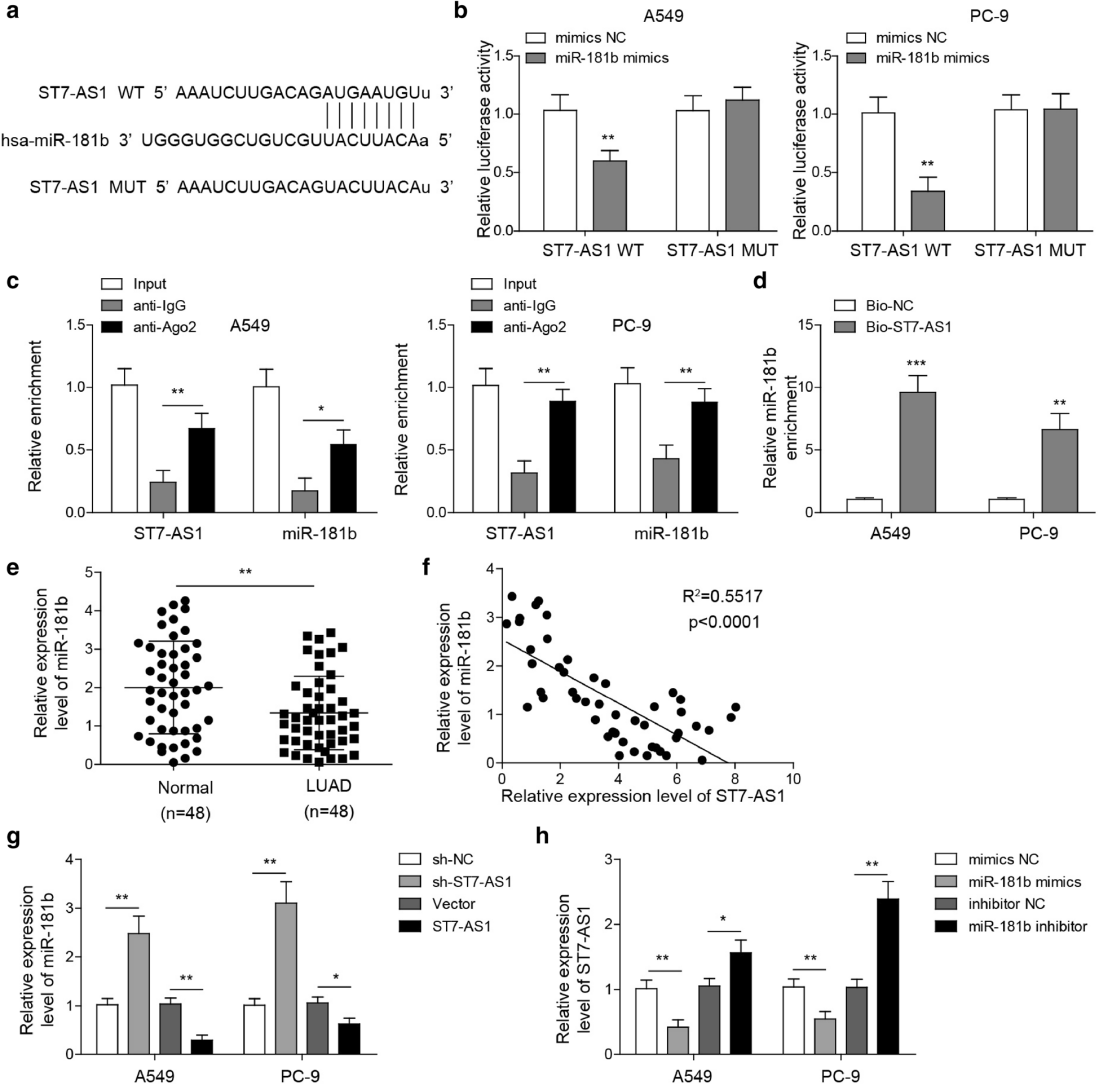
ST7-AS1 directly targets miR-181b-5p. **a **StarBase software revealed the potential binding sequences between miR-181b-5p and ST7-AS1. **b** Either WT or MUT binding sequence of ST7-AS1 was cloned into luciferase vector, and co-transfected into A549 and PC-9 cells with mimics NC or miR-181b-5p mimics. The reporter activity was measured by luciferase assay. **c** The interaction of ST7-AS1 or miR-181b-5p with Ago2 from A549 and PC-9 cells was examined by RIP assay. **d** ST7-AS1 binding sequence was prepared into biotin-labelled probe and incubated with whole cell lysate from A549 and PC-9 cells. miR-181b-5p pulled down by the probe was detected by RT-qPCR. **e** The expression of miR-181b-5p was measured by RT-qPCR and compared between 48 pairs of LUAD tissues and normal lung tissues. **f** Correlation analysis revealed negative correlation between miR-181b-5p and ST7-AS1 in LUAD tissues. **g** The expression of miR-181b-5p after ST7-AS1 knockdown or overexpression in A549 and PC-9 cells was measured by RT-qPCR. **h** The expression of ST7-AS1 after transfection of A549 and PC-9 cells with miR-181b-5p mimics or inhibitor was measured by RT-qPCR. *P < 0.05; **P < 0.01; ***P < 0.001

A common mechanism responsible for lncRNA activities is by functioning as a decoy or sponge to miRNAs. To investigate the specific molecular mechanisms underlying the biological effects of lncRNA ST7-AS1, we performed bioinformatic analysis using starBase platform (http://starbase.sysu.edu.cn/) and identified miR-181b-5p as a potential miRNA that interacts with ST7-AS1 (Fig. 3a). To determine whether ST7-AS1 directly regulated miR-181b-5p, we clone either wild-type (WT) or mutated (MUT) binding sequence of ST7-AS1 into a luciferase reporter vector and co-transfected with miR-181b-5p mimics or mimics NC into PC-9 and A549 cells. Figure 3b showed that miR-181b-5p mimics specifically suppressed the luciferase activity driven by the WT, but not MUT ST7-AS1, in both PC-9 and A549 cells. Furthermore, RIP assay revealed the presence of both ST7-AS1 and miR-181b-5p in the same complex as Ago2, the catalytic component in the RNA-induced silencing complex [25] (Fig. 3c). RNA pulldown assay using biotin-labelled probe containing the ST7-AS1 binding sequence revealed its capability to pull down miR-181b-5p from extracts of both PC-9 and A549 cells (Fig. 3d). Together, these data suggest a direct interaction and a negative regulation between ST7-AS1 and miR-181b-5p at the predicted binding site. Next, we compared the expression of miR-181b-5p between 48 pairs of LUAD tissues and normal tissues. As shown in Fig. 3e, miR-181b-5p was significantly down-regulated in LUAD tissues. Correlation analysis revealed significant negative association between ST7-AS1 and miR-181b-5p levels in LUAD tissues (Fig. 3f). To examine the regulation between ST7-AS1 and miR-181b-5p, we applied both loss-of-function and gain-of-function strategies. As shown in Fig. 3g, sh-ST7-AS1 significantly up-regulated while ST7-AS1 potently down-regulated miR-181b-5p expression in both PC-9 and A549 cells. Reciprocally, miR-181b-5p mimics was sufficient to reduce, while miR-181b-5p inhibitor markedly increased ST7-AS1 mRNA (Fig. 3h). Therefore, through direct interaction, ST7-AS1 and miR-181b-5p mutually and negatively regulate the expression of one another in LUAD cells, which may account for their negative correlation in tumour tissues.

### MiR-181b-5p critically controls the viability, EMT, migration and invasion of LUAD cells

**Fig. 4:**
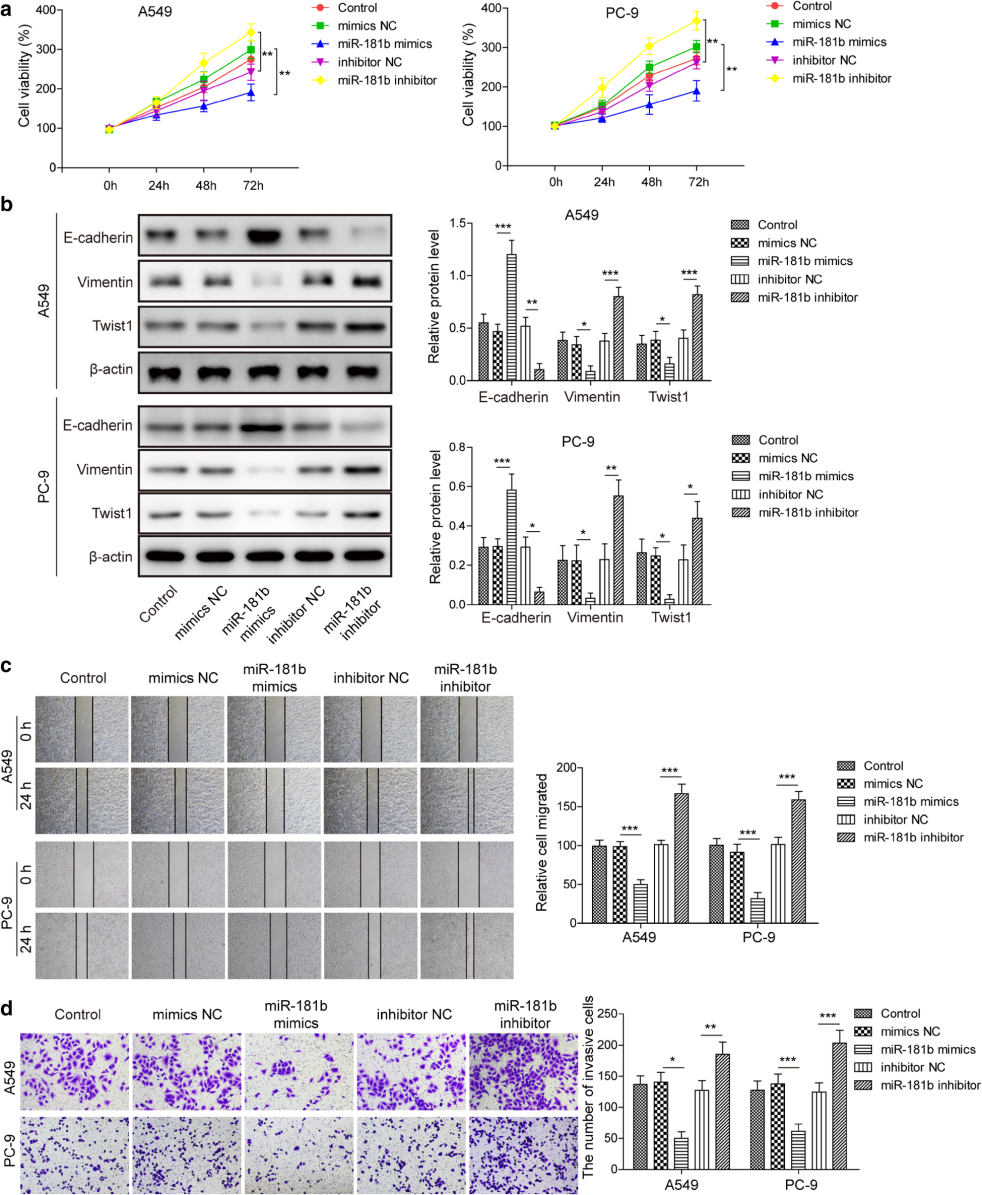
miR-181b-5p suppresses malignant phenotypes in LUAD cells. A549 and PC-9 cells were either non-transfected (Control) or transfected with mimics NC, miR-181b-5p mimics, inhibitor NC, or miR-181b-5p inhibitor. **a** Cell viability of indicated cells was measured by MTT assay at 24 and 48 h after transfection, respectively. **b** The protein levels of EMT-related biomarkers, E-cadherin, vimentin, and Twist1 in indicated A549 and PC-9 cells were measured by Western blot assay, with representative Western image shown on the top and protein quantification on the bottom. **c** The migration of indicated A549 and PC-9 cells was assessed by wound healing assay. **d** The invasion of indicated A549 and PC-9 cells was assessed by Transwell assay. Representative images of invaded cells shown on the left and the number of invaded cells quantified on the right. *P < 0.05; **P < 0.01; ***P < 0.001

To understand the biological importance of miR-181b-5p in LUAD, we transfected PC-9 and A549 cells with miR-181b-5p mimics or miR-181b-5p inhibitor to alter its level in these cells. Viability assay showed that miR-181b-5p mimics significantly reduced, while miR-181b-5p inhibitor markedly boosted the viability of both cells, when compared to the corresponding mimics NC and inhibitor NC cells (Fig. 4a). Assays on EMT-related biomarkers showed that overexpressing miR-181b-5p with miR-181b-5p mimics was sufficient to up-regulate E-cadherin and down-regulate vimentin and Twist1 in both PC-9 and A549 cells, and thus inhibiting EMT. MiR-181b-5p inhibitor acted oppositely to promote EMT (Fig. 4b). In addition, miR-181b-5p mimics decreased migration while miR-181b-5p inhibitor induced cell migration (Fig. 4c) and invasion (Fig. 4d), where miR-181b-5p mimics reduced while miR-181b-5p inhibitor enhanced the migration and invasion of both PC-9 and A549 cells. Therefore, miR-181b-5p essentially regulated the viability, EMT, and migration/invasion of LUAD cells.

### MiR-181b-5p directly targets KPNA4

**Fig. 5:**
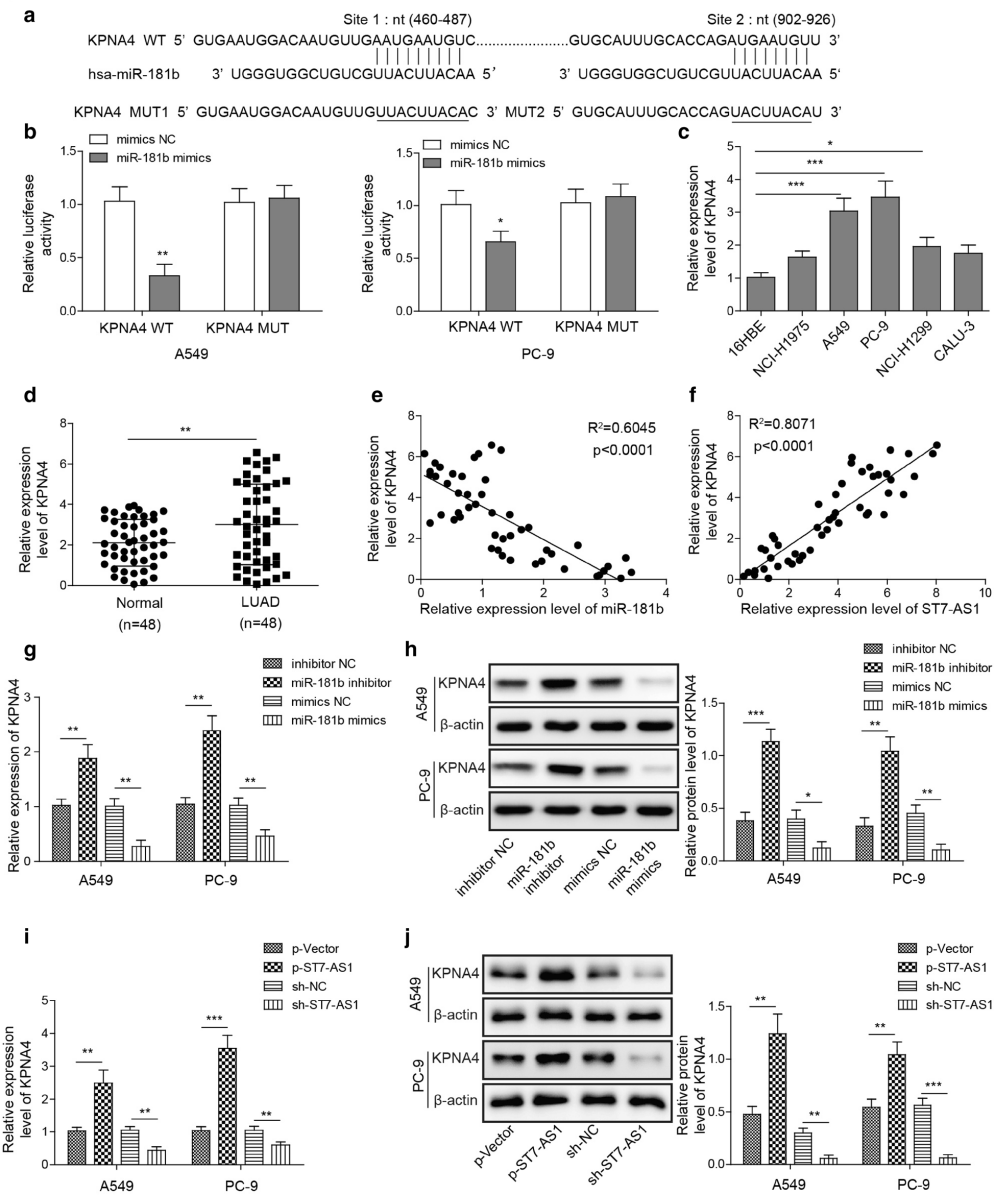
Mir-181b-5p directly targets KPNA4 while ST7-AS1 upregulates it. **a** Bioinformatic analysis revealed two potential binding sites between miR-181b-5p and KPNA4. **b** Either WT or MUT binding sequence of KPNA4 was cloned into luciferase vector, and co-transfected into A549 and PC-9 cells with mimics NC or miR-181b-5p mimics. The reporter activity was measured by luciferase assay. **c** The expression of KPNA4 in indicated cell lines was measured by RT-qPCR. **d** The expression of KPNA4 was measured by RT-qPCR and compared between 48 pairs of LUAD tissues and normal lung tissues. Correlation analysis showed negative correlation between KPNA4 and miR-181b-5p (**e**), and positive correlation between KPNA4 and ST7-AS1 (**f**) in LUAD tissues. The expression of KPNA4 in A549 and PC-9 cells transfected with mimics NC, miR-181b-5p mimics, inhibitor NC, or miR-181b-5p inhibitor was measured on the mRNA level by RT-qPCR (**g**) and on the protein level by Western blot assay. **h** The expression of KPNA4 in A549 and PC-9 cells transfected with sh-NC, sh-ST7-AS1, p-Vector, or p-ST7-AS1 was measured on the mRNA level by RT-qPCR (**i**) and on the protein level by Western blot assay (**j**). *P < 0.05; **P < 0.01; ***P < 0.001

It was reported that KPNA4 is a direct target and antagonizes the EMT-inhibiting effect of miR-181b-5p in glioblastoma [26]. We set out to examine whether KPNA4 was also the target of miR-181b-5p in LUAD cells. First, bioinformatic analysis revealed two potential binding sites in KPNA4 gene that may target by miR-181b-5p (Fig. 5a). Luciferase assay showed that miR-181b-5p mimics specifically inhibited the luciferase gene expression containing the WT, but not MUT KPNA4 binding sites (Fig. 5b). Next, by examining the expression level of KPNA4 mRNA in different LUAD cells (NCI-H1975, NCI-H1299, A549, PC-9 and CALU-3), we found that, similar to the expression pattern of ST7-AS1, KPNA4 was significantly up-regulated in PC-9 and A549, but not other LUAD cells, when compared to 16HBE cells (Fig. 5c). Similarly, KPNA4 level was also highly enhanced in LUAD tissues, when compared to normal tissues (Fig. 5d). Correlation analysis showed that KPNA4 level was negative associated with that of miR-181b-5p (Fig. 5e), but positively with ST7-AS1 (Fig. 5f) in LUAD tissues. In both PC-9 and A549 cells, miR-181b-5p mimics was sufficient to reduced, while miR-181b-5p inhibitor up-regulated KPNA4 level, both on the mRNA (Fig. 5g) and the protein (Fig. 5h) levels. In contrast, knocking down ST7-AS1 with sh-ST7-AS1 reduced, while overexpressing ST7-AS1 up-regulated KPNA4 mRNA (Fig. 5i) and protein (Fig. 5j). Taken together, these data suggest that miR-181b-5p directly targets KPNA4, and ST7-AS1, by sponging miR-181b-5p, up-regulates KPNA4. This regulatory mechanism was supported by the positive correlation between KPNA4 and ST7-AS1, as well as the negative one between KPNA4 and miR-181b-5p in LUAD tissues.

### ST7-AS1 promotes malignancy of LUAD cells via the miR-181b-5p/KPNA4 axis

**Fig. 6:**
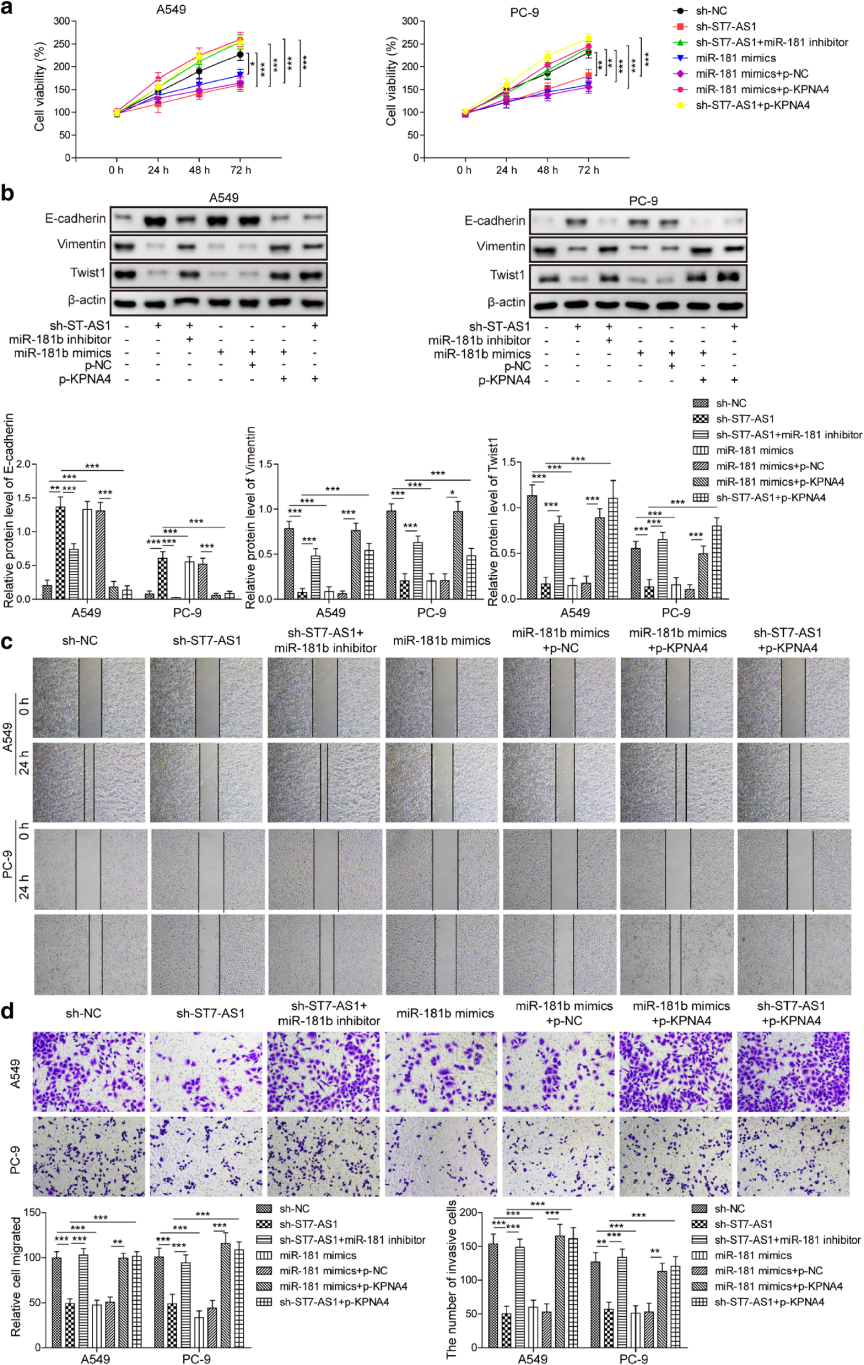
The miR-181b-5p/KPNA4 axis essentially mediated the oncogenic activities of ST7-AS1. A549 and PC-9 cells were transfected as indicated to alter the expressions of ST7-AS1, miR-181b-5p and/or KPNA4. **a** Cell viability of indicated cells was measured by MTT assay at 24 h and 48 h after transfection, respectively. **b** The protein levels of EMT-related biomarkers, E-cadherin, vimentin, and Twist1 in indicated A549 and PC-9 cells were measured by Western blot assay, with representative Western image shown on the top and the quantification of relative protein expression on the bottom. **c** The migration of indicated A549 and PC-9 cells was assessed by wound healing migration assay. **d **The invasion of indicated A549 and PC-9 cells was assessed by Transwell assay, with representative images of invaded cells shown on the left and the number of invaded cells quantified on the right. *P < 0.05; **P < 0.01; ***P < 0.001

To test whether ST7-AS1 regulates malignant phenotypes of LUAD cells through the miR-181b-5p/KPNA4 axis, we transfected miR-181b-5p inhibitor or overexpressed KPNA4 in sh-ST7-AS1 cells, or overexpressed KPNA4 in cells transfected with miR-181b-5p mimics. As observed earlier, sh-ST7-AS1 cells and miR-181b-5p mimics-transfected cells presented significantly reduced cell viability, applying miR-181b-5p inhibitor in sh-ST7-AS1 cells or overexpressing KPNA4 in either sh-ST7-AS1 or miR-181b-5p mimics-transfected (both PC-9 and A549) cells was sufficient to rescue the viability of these cells (Fig. 6a). Similarly, transfecting miR-181b-5p inhibitor to sh-ST7-AS1 cells or overexpressing KPNA4 in sh-ST7-AS1 or miR-181b-5p mimics-transfected (both PC-9 and A549) cells reversed the inhibitory effects of targeting ST7-AS1 or overexpressing miR-181b-5p on EMT (Fig. 6b), cell migration (Fig. 6c), and cell invasion (Fig. 6d and e), suggesting that targeting miR-181b-5p and up-regulating KPNA4 critically mediate the pro-malignancy of ST7-AS1.

### Targeting ST7-AS1 inhibited xenograft growth in vivo

**Fig. 7:**
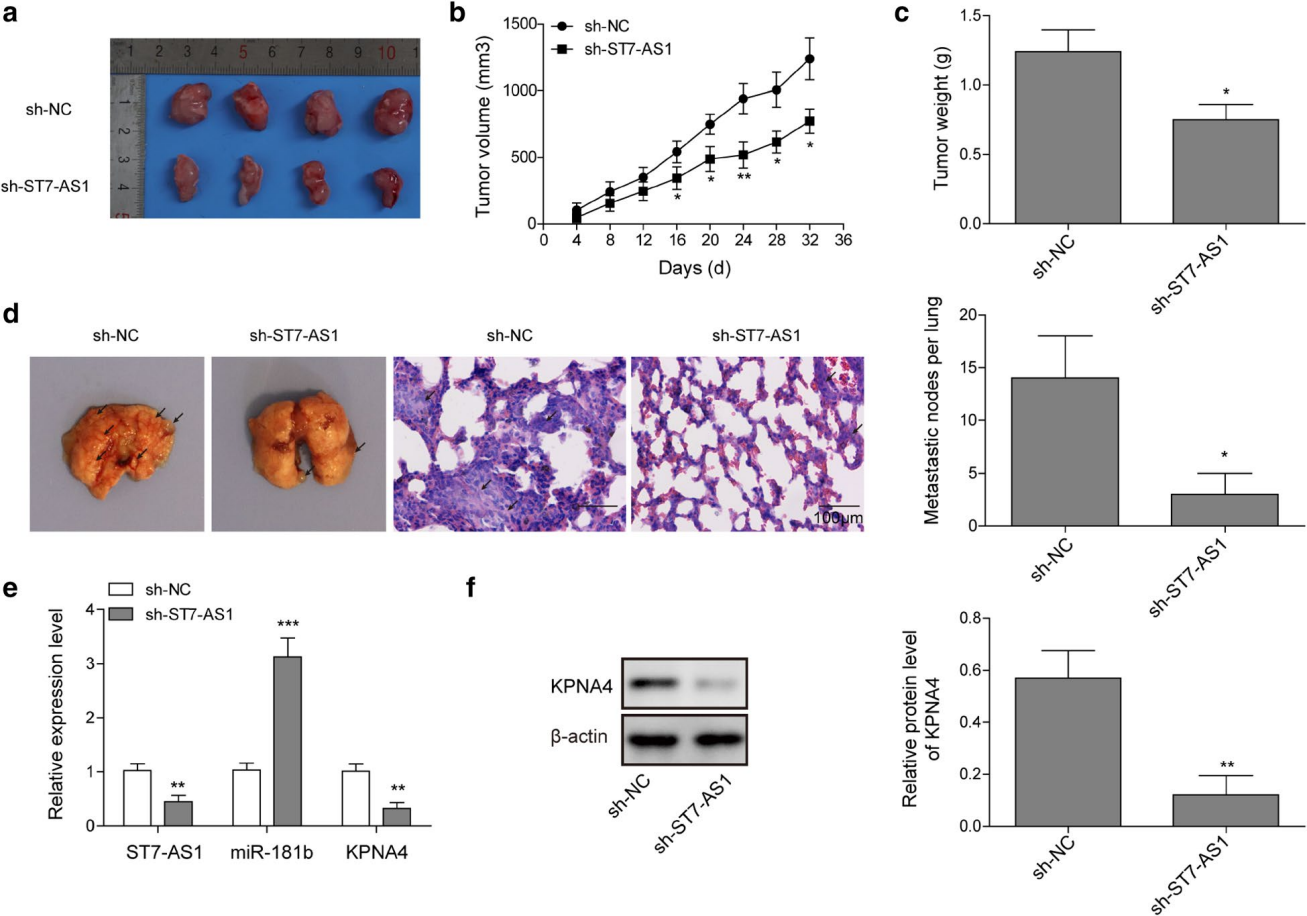
Targeting ST7-AS1 inhibited xenograft growth in vivo. Sh-NC vs. sh-ST7-AS1 PC-9 cells were subcutaneously injected into nude mice (n = 4/group). The picture (**a**), the growth curve (**b**), and the weight (**c**) of xenografts were compared between the two groups. **d** Sh-NC vs. sh-ST7-AS1 PC-9 cells were injected into nude mice through the tail vein (n = 4/group). On day 30, the metastatic foci (arrows) in the lung tissues were imaged (upper panels) and analyzed by HE staining (lower panels). The expressions of ST7-AS1, miR-181b-5p, and KPNA4 mRNAs were examined by RT-qPCR (**e**) and that of KPNA4 protein by Western blot (**f**) in xenografts. *P < 0.05; **P < 0.01; ***P < 0.001

Lastly, we examined the significance of our in vitro findings using an in vivo xenograft model of PC-9 cells. We subcutaneously inoculated sh-NC- vs. sh-ST7-AS1-transfected PC-9 cells into nude mice (n = 4/group). We found that sh-ST7-AS1 cells generated significantly smaller xenografts (Fig. 7a), consistent with their growth curve in vivo (Fig. 7b) and their weights (Fig. 7c). Furthermore, we also examined the impact of targeting ST7-AS1 on the metastatic spread of PC-9 cells using an experimental metastasis model. Upon injecting sh-NC- vs. sh-ST7-AS1-transfected PC-9 cells into the tail vein (n = 4/group), we detected significantly fewer metastatic foci developed in the lung tissue (Fig. 7d), suggesting that knocking down ST7-AS1 inhibits not only xenograft growth but also the metastasis of PC-9 cells in vivo*.* Expression analysis of the xenografts showed that ST7-AS1 was significantly down-regulated in sh-ST7-AS1 xenografts, suggesting the successful and sustained knockdown of ST7-AS1. Concomitantly, miR-181b-5p was significantly up-regulated (Fig. 7e), while KPNA4 reduced on both mRNA (Fig. 7e) and protein levels (Fig. 7f) in sh-ST7-AS1 xenografts, supporting the association between the anti-cancer activity of sh-ST7-AS1 and the up-regulation of miR-181b-5p as well as the down-regulation of KPNA4.

## Discussion

Lung cancer is the most lethal cancer worldwide, and LUAD is the major subtype of lung cancer [27]. Although great progress has been made in surgical resection, chemoradiotherapy or drugs specifically targeting metastasis and recurrence, the prognosis of lung cancer patients remains poor [9]. Hence, it is of significant clinical importance to uncover the molecular mechanisms responsible for carcinogenesis and identify tumour biomarkers for predicting the prognosis of LUAD.

Although a newly identified lncRNA, studies have suggested the oncogenic feature of ST7-AS1 in at least gastric cancer and laryngeal squamous cell carcinoma [11, 12]. Not only its expression up-regulated in tumour tissues from gastric cancer and LSCC, but its higher expression associated with tumours of advanced stages or positive for metastasis or indicating poor survival of cancer patients [11, 12]. Similarly, here we showed that ST7-AS1 was significantly up-regulated in LUAD tissues, associated with tumours positive for lymph node metastasis or of higher TNM stages, and correlated with shorter overall survival of LUAD patients. These data strongly demonstrated the value of ST7-AS1 as a biomarker for advanced LUAD or those with poor prognosis and suggested the potential involvement of ST7-AS1 in LUAD progression. To understand the biological functions of ST7-AS1, we compared its expression between five different LUAD cell lines and normal bronchial epithelial cells 16HBE. To our surprise, we only detected significant up-regulation of ST7-AS1 in PC-9 and A549 cells, but not in NCI-H1975, NCI-H1299, or CALU-3 cells, suggesting the heterogeneity of different LUAD cell lines and non-ubiquitous requirement of ST7-AS1 for LUAD development. Functionally, we showed that ST7-AS1 was essential for maintaining the viability, EMT, migration, and invasion of LUAD cells, consistent with its association to highly malignant LUAD and with its functions reported in other cancers [11, 12]. Collectively, these finding support the oncogenic activities of ST7-AS1 in LUAD, which was further corroborated with the in vivo finding that knocking down ST7-AS1 inhibited xenograft growth. In addition, we also showed that knocking down ST7-AS1 suppressed experimental metastasis of LUAD cells to the lung, consistent with the finding that higher ST7-AS1 level was associated with tumour of higher malignancy and metastasis.

Besides lncRNA, emerging evidence also indicates that miRNAs may present either oncogenic or tumour suppressive activities in almost all kind of tumours, including lung cancer [28]. It was reported that miR-181b-5p inhibited chemoresistance in cisplatin-resistant H446 small-cell lung cancer cells by targeting Bcl-2 [29]. Moreover, miR-181b-5p regulated metastasis by targeting TGF-β/Smad signalling pathway was downregulated in NSCLC and inhibited cell motility by directly targeting HMGB1 [30]. Consistent with previous reports, this study revealed that miR-181b-5p inhibits EMT in LUAD cells, indicating that miR-181b-5p could be a potential molecular target of LUAD.

Recently, a new regulatory molecular mechanism of lncRNA, called ceRNA, was reported whereby an lncRNA may function as a miRNA sponge to modulate the expression of miRNA targets [31]. Previous researches reported that miR-181b-5p is down-regulated in NSCLC [21], as well as in pancreatic cancer, glioblastoma [30, 32, 33]. Therefore, we speculated that miR-181b-5p may play a potential regulatory role of EMT in LUAD. In this study, we indicated that lncRNA ST7-AS1 directly bound to miR-181b-5p and functioned as a miRNA sponge to trap miR-181b-5p, therefore up-regulating miR-181b-5p target, KPNA4 in LUAD cells. The miRNA sponge function of ST7-AS1 was supported by a series of experimental findings. First, sh-ST7-AS1 upregulated miR-181b-5p while overexpressing ST7-AS1 down-regulated miR-181b-5p. Second, luciferase reporter, RIP, and RNA pull-down assays confirmed the direct and specific binding between ST7-AS1 and miR-181b-5p. Our results supported that ST7-AS1 is a sponge of miR-181b-5p in LUAD.

Moreover, our data revealed that the downregulation of KPNA4 by sh-ST7-AS1 was rescued by inhibiting miR-181b-5p. KPNA4 mediates TNF-α-induced NF-κB p50/p65 nuclear import [34]. KPNA4 is also required for the activation of Notch signalling, which is associated with tumour recurrence as well as tumour initiation and growth [35]. However, there are still limited reports on its regulatory roles in tumours. It was reported that inhibiting KPNA4 attenuated prostate cancer metastasis [36]. Here, consistent with previous reports, we demonstrated that ST7-AS1 promoted multiple malignant phenotypes of LUAD cells by sponging miR-181b-5p, and thus up-regulating KPNA4, which also indicated that KPNA4 could be a potential target for cancer therapy.

## Conclusions

Overall, we evidenced the oncogenic role of lncRNA ST7-AS1 in LUAD, the overexpression of which promoted viability, EMT, migration and invasion of cancer cells via sponging miR-181b-5p, and subsequently releasing its suppression on KPNA4. These results unveiled important pathogenic mechanisms of LUAD and provided potential targets for LUAD therapy.

## Data Availability

All data generated or analyzed during this study are included in this published article [and its supplementary information files].

## Abbreviations

ST7-AS1: Suppressor of tumorigenicity 7 antisense RNA 1
qRT-PCR: Quantitative real-time polymerase chain reaction
EMT: Epithelial-to-mesenchymal transition
NSCLC: Non-small cell lung cancer
LUAD: Lung adenocarcinoma
lncRNAs: Long non-coding RNAs
3′UTR: 3′Untranslated region
TGF-β1: Transforming growth factor-β1
ceRNA: Competing endogenous RNA
HSCR: Hirschsprung disease
MiRNAs: MicroRNAs
